# Lovastatin enhances adenovirus-mediated TRAIL induced apoptosis by depleting cholesterol of lipid rafts and affecting CAR and death receptor expression of prostate cancer cells

**DOI:** 10.18632/oncotarget.3073

**Published:** 2014-12-26

**Authors:** Youhong Liu, Lin Chen, Zhicheng Gong, Liangfang Shen, Chinghai Kao, Janet M. Hock, Lunquan Sun, Xiong Li

**Affiliations:** ^1^ Center for Molecular Medicine, Xiangya Hospital, Central South University, Changsha, Hunan, China; ^2^ Department of Pharmacy, Xiangya Hospital, Central South University, Changsha, Hunan, China; ^3^ Department of Oncology, Xiangya Hospital, Central South University, Changsha, Hunan, China; ^4^ Department of Urology, Indiana University School of Medicine, Indianapolis, IN, USA; ^5^ The Polis Center, Indiana University and Purdue University at Indianapolis, Indianapolis, IN, USA; ^6^ Department of Integrated Traditional Chinese and Western Medicine, Xiangya Hospital, Central South University, Changsha, Hunan, China

**Keywords:** Statins, Adenovirus, Gene Therapy, TRAIL, Apoptosis

## Abstract

Oncolytic adenovirus and apoptosis inducer TRAIL are promising cancer therapies. Their antitumor efficacy, when used as single agents, is limited. Oncolytic adenoviruses have low infection activity, and cancer cells develop resistance to TRAIL-induced apoptosis. Here, we explored combining prostate-restricted replication competent adenovirus-mediated TRAIL (PRRA-TRAIL) with lovastatin, a commonly used cholesterol-lowering drug, as a potential therapy for advanced prostate cancer (PCa). Lovastatin significantly enhanced the efficacy of PRRA-TRAIL by promoting the *in vivo* tumor suppression, and the *in vitro* cell killing and apoptosis induction, via integration of multiple molecular mechanisms. Lovastatin enhanced PRRA replication and virus-delivered transgene expression by increasing the expression levels of CAR and integrins, which are critical for adenovirus 5 binding and internalization. Lovastatin enhanced TRAIL-induced apoptosis by increasing death receptor DR4 expression. These multiple effects of lovastatin on CAR, integrins and DR4 expression were closely associated with cholesterol-depletion in lipid rafts. These studies, for the first time, show correlations between cholesterol/lipid rafts, oncolytic adenovirus infection efficiency and the antitumor efficacy of TRAIL at the cellular level. This work enhances our understanding of the molecular mechanisms that support use of lovastatin, in combination with PRRA-TRAIL, as a candidate strategy to treat human refractory prostate cancer in the future.

## INTRODUCTION

Prostate cancer (PCa) is the most commonly diagnosed, noncutaneous malignancy, and the second leading cause of cancer death, in males in the United States. Approximately 233,000 new diagnoses and 29,480 deaths are predicted to occur in 2014 [1]. Patients frequently exhibit locally advanced disease and/or detectable distant bone metastases at initial presentation. Androgen ablation remains the main treatment modality recommended for patients with advanced disease, with an emerging role for chemotherapy. However, hormonal ablation is not curative. PCa inevitably progresses to an androgen-independent (AI) lethal phenotype over time. No curative therapy is available to treat PCa after it becomes hormone refractory and metastasizes to bone. At this point, the disease becomes fatal.

Gene therapy is one option that holds promise to improve the targeted killing of hormone refractory PCa cells. For example, adenovirus 5 (Ad) vectors have a broad host range and can infect both normal and tumor cells [2, 3]. To reduce unwanted side effects, it is important to develop oncolytic tissue and tumor-restricted, replication-competent adenoviral vectors (TRRA). TRRA exhibit superior antitumor efficacy and safety when compared to replication-deficient Ad vectors, because TRRA actively propagates in and lyses the targeted cancer cells. In contrast, TRRA replication activity is low in normal cells. We developed a prostate-restricted, replication competent adenoviral vector (PRRA) by placing both adenoviral *E1a* and *E4* genes under the control of a PSES enhancer to direct viral replication in a tissue and tumor-specific manner [4]. PSES is a chimeric prostate-specific enhancer sequence, which combines the enhancer elements from PSA and PSMA genes, two well-studied prostate-specific biomarkers. PSES demonstrated high tumor specific activity in PSA/PSMA positive PCa cell lines [5]. PRRA showed prostate-restricted replication and killing activities in PSA/PSMA positive PCa cell lines [4]. However, the low virus infection efficiency and the limited virus distribution in the solid tumors limit the therapeutic potential of these oncolytic PRRAs for applications in prostate cancer.

To improve therapeutic efficacy, we developed a series of gene-armed PRRAs by delivering suicide gene HSV-TK [6], apoptosis inducer TRAIL [7] and FasL [8], angiogenesis inhibitor endostatin and angiostatin fusion gene [9] and antitumor immune stimulator IL-12 [10]. The cancer-selective death-inducing character of TRAIL makes it an attractive candidate molecule for cancer therapy. TRAIL induces receptor-mediated apoptosis in a wide variety of cancer cell lines of diverse origin. TRAIL binding to death domain-controlled receptors, DR4 and DR5, triggers the death-inducing signal complex (DISC) formation and activation of procaspase-8, which in turn activates caspase-3, leading to cell death [11]. Normal cells can escape TRAIL-induced apoptosis through the expression of an antagonist decoy receptor, TRID [12]. A challenge to the *in vivo* use of TRAIL is that some cancer cells are resistant to TRAIL treatment. Many molecules in the TRAIL signaling pathway, including FLIPs, IAPs and IG20, can contribute to resistance mechanisms [13]. This means that high concentration of TRAIL protein is an essential prerequisite for this therapy to be viable [14]. We developed a TRAIL-expressing PRRA to improve delivery and targeting of TRAIL to tumor sites. PRRA-TRAIL improved the antitumor efficacy of both PRRA and TRAIL by activating multiple molecular mechanisms [7]. Importantly, the PRRA-TRAIL virus-infected tumor cells produced soluble TRAIL, which triggered apoptosis of the surrounding cells uninfected by viruses [7].

An alternative strategy to increase tumor cell killing is to combine pharmaceutical agents with gene therapy. Pharmacologic agents that may be useful in this regard are the statins, 3-hydroxy-3-methylglutaryl (HMG) CoA reductase inhibitors, that are commonly used to lower cholesterol. Several large population-based epidemiological studies suggest that lovastatin reduced the risk of PCa [15-17]. Statins exert antitumor effects on PCa cell lines by inhibiting cell proliferation [18], interfering with the cell cycle [19] and inducing apoptosis [20]. Lovastatin molecular mechanisms include increased cytochrome c release, which reduced pro-caspase-3 and increased activated caspase-3, independently of P53-induced apoptosis when combined with other chemotherapeutics, lovastatin exerts a synergistic effect to suppress tumor growth [21-23].

Here, we explored the consequences of combining lovastatin with PRRA-mediated TRAIL in proof-of-principle experiments to support development of a novel strategy to treat refractory PCa. We determined the *in vivo* antitumor efficacy and extent of *in vitro* cell killing and apoptosis induction of PRRA-TRAIL and lovastatin therapy. Viral replication activity and transgene expression were assessed. Viral binding, internalization and intercellular trafficking were monitored after PCa cells were pre-treated with lovastatin. The levels of cholesterol/lipid rafts on cellular membranes were assessed. Induction of apoptosis by either lovastatin or TRAIL alone or the combination of treatments was evaluated. The correlation of cancer cell apoptosis induced by lovastatin with the level of cholesterol/lipid rafts was analyzed. The expression of adenovirus-associated receptors CAR, selected integrins and the death receptors, DR4 and DR5, were assessed after lovastatin treatment. These studies add to our understanding of the role of membrane cholesterol in oncolytic adenovirus infection efficiency, and in induction of apoptosis by TRAIL. In summary, we identified key molecular mechanisms that support use of lovastatin in combination with PRRA-TRAIL as a candidate strategy to treat refractory PCa.

## RESULTS

### Lovastatin significantly enhanced *in vivo* antitumor efficacy of oncolytic PRRA AdE4 and AdE4-TRAIL

**Figure 1:**
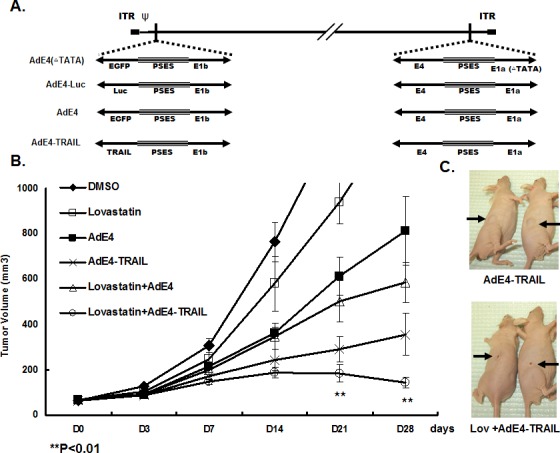

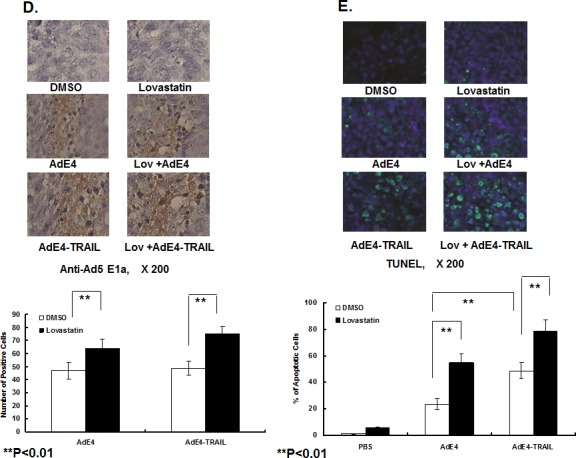
Lovastatin significantly enhanced tumor suppression efficacy of oncolytic adenovirus constructs, AdE4 and AdE4-TRAIL A. Structure diagram of prostate cancer-specific replicative adenovirus AdE4, AdE4-TRAIL, AdE4-Luc (AdE4 vector encoding a luciferase gene) and AdE4(ΔTATA) (a replication-deficient adenovirus due to the deletion of TATA box of Ad *E1a* gene). B. CWR22rv xenografts were established in athymic nude mice. Mice were randomized 3 weeks after cell inoculation (8 mice/group, 1 tumor xenograft/ mouse). Tumors were treated with lovastatin at 15 mg/kg/day, i.p. for 7 consecutive days), or AdE4 (2 ×10^7^ IFU in 100mL 1×PBS i.t.), AdE4-TRAIL (2 ×10^7^ IFU in 100mL 1×PBS i.t.), or lovastatin (i.p.) combined with AdE4 (i.t.), or lovastatin (i.p.) combined with AdE4-TRAIL (i.t.), for up to 28 days. Tumor size was measured on day 3 (D3), and then once every 7 days until 28 days (D28), and expressed as the average per group (n=8 xenografts/group),. C. Representative photographs of tumor xenografts (arrows) in 2 of the mice treated with AdE4-TRAIL (top) or lovastatin plus AdE4-TRAIL (bottom). On day 28, gross tumor size is much less in mice treated with lovastatin plus AdE4-TRAIL compared to those in mice treated with AdE4-TRAIL alone. D. Photomicrographs of immunohistochemistry of virus (Ad E1a) in tissue sections of xenografts after 28 days of treatment. The number of positive cells was counted in 10 randomly selected vision fields (original magnification: ×200) for each tissue section; the average of 3 tissue sections was used to represent each tumor. Lovastatin significantly enhanced virus infection inside tumor tissues (see graph below photomicrograph). E. Photomicrographs of tissue sections of xenografts showing fluorescence-labeled TUNEL+ apoptotic cells in tumor xenografts. The green fluorescent cells were counted in 10 randomly selected vision fields (original magnification: ×200) for each tissue section; the average of 3 tissue sections was used to represent each tumor. Lovastatin significantly enhanced AdE4-TRAIL-induced apoptosis inside tumor tissues (see graph below photomicrograph).

In previous work, we developed a prostate-restricted replication competent adenoviral (PRRA) vector AdE4PSESE1a (AdE4), in which both adenoviral *E1a* and *E4* genes were controlled by PSES enhancer [4]. The adenoviral vector was used to deliver a series of therapeutic genes such as HSV-TK [6], TRAIL [7], FasL [8] and endostatin-angiostatin fusion genes [9] by replacing EGFP gene with therapeutic genes (Figure 1A) to improve antitumor efficacy. Here, we first tested if lovastatin improved the *in vivo* antitumor efficacy of AdE4 or AdE4-TRAIL in PSA/PSMA positive, androgen-independent CWR22rv subcutaneous tumor xenografts in nude mice. In contrast to DMSO control, lovastatin alone, injected i.p. at 15 mg/kg/day, for 7 consecutive days, delayed the growth of tumor xenografts. Consistent with previous reports, AdE4 inhibited tumor growth, but only for the first 14 days after virus injection. After 14 days, the tumors grew exponentially [4]. Lovastatin significantly enhanced the antitumor efficacy of AdE4. Average tumor size decreased by approximately 20% on day 21, and by 40% on day 28 (P<0.01). AdE4-TRAIL exhibited better antitumor efficacy than AdE4, due to incorporation of TRAIL (P<0.01). Interestingly, lovastatin greatly improved the antitumor efficacy of AdE4-TRAIL (P<0.01), because the tumor burden remained decreased for more than 14 days after virus injection (Figure 1B). On day 28, gross tumor size was much less in mice treated with lovastatin plus AdE4-TRAIL, than in those treated with AdE4-TRAIL alone (Figure 1B).

We harvested tumor xenografts at 28 days after cell incubation, and tested for adenovirus infection inside tumor tissues. No significant difference in adenovirus infection efficiency was observed between AdE4 and AdE4-TRAIL-treated tumors. When compared to control AdE4-infected tumors, lovastatin significantly increased AdE4 or AdE4-TRAIL viral-infection efficiency (P<0.01, Figure 1D). We also analyzed cell apoptosis inside tumor tissues by using an *in situ* fluorescent TUNEL assay. In contrast to DMSO control, lovastatin or AdE4 alone induced apoptosis in some cells. When combined with AdE4, lovastatin increased cell apoptosis (P<0.01). As predicted, AdE4-TRAIL induced more cell apoptosis than AdE4, due to the added apoptosis induction effect of TRAIL (P<0.01). Lovastatin further enhanced AdE4-TRAIL-induced apoptosis (P<0.01, Figure 1E). These data suggest that lovastatin significantly enhances the oncolytic effect of replication competent adenoviruses, and may also enhance TRAIL-induced apoptosis.

### Lovastatin enhanced *in vitro* cell killing and apoptosis induction by AdE4 and AdE4-TRAIL in PCa cells

**Figure 2:**
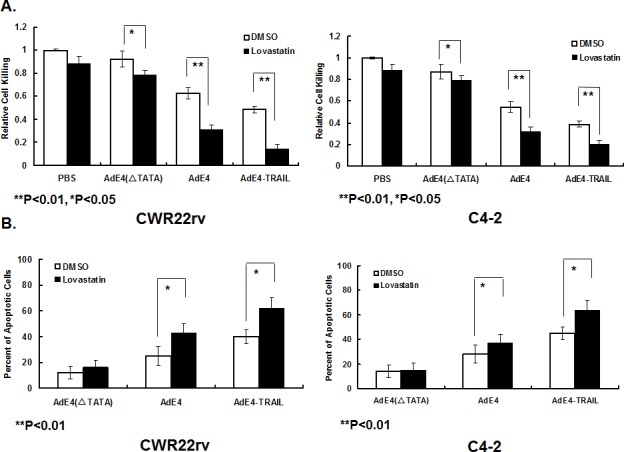
Lovastatin significantly enhanced induction of cell killing and apoptosis by AdE4 and AdE4-TRAIL CWR22rv and C4-2 cells were treated with lovastatin (5μM for CWR22rv and 2.5μM for C4-2) for 16 hours, followed by virus infection of AdE4(ΔTATA)(replication-deficient adenovirus), AdE4 or AdE4-TRAIL (100 vp/cell). A. The cells were stained with crystal violet, and the optical density was analyzed at OD490, at day 5 after virus infection. An index of relative cell killing activity for each treatment was calculated by comparing the treatment groups to control (PBS+DMSO). B. The percent of apoptotic cells was calculated at 48 hours after virus infection, as described in the Methods.

As lovastatin significantly enhanced *in vivo* antitumor efficacy of both AdE4 and AdE4-TRAIL, we tested if lovastatin would improve the *in vitro* killing effects of AdE4 and AdE4-TRAIL in PSA/PSMA-positive PCa cells. CWR22rv or C4-2 cells were treated with either lovastatin, AdE4 or AdE4-TRAIL alone, or in combination. The doses of lovastatin and oncolytic viruses used in the combination experiments were determined in preliminary studies (data not shown). As shown in Figure 2A, the control construct, AdE4(ΔTATA)(100 vp/cell), did not exhibit detectable cell killing in CWR22rv cells, because the TATA box of *E1a* gene was deleted and the virus is replication-deficient (Figure 1A). Lovastatin (5 μM) alone slightly increased cell killing, when compared to DMSO control (P<0.05). AdE4 (100 vp/cell) exhibited some cell killing activity because of the oncolytic effect of replication competent adenovirus. Lovastatin (5 μM) significantly increased the cell killing activity of AdE4 (100 vp/cell) (P<0.01). Furthermore, AdE4-TRAIL exhibited more cell killing activity than AdE4 (P<0.01). Lovastatin further enhanced the cell killing activity of AdE4-TRAIL (P<0.01).

Similar results were observed in C4-2 cells. The control, AdE4(ΔTATA) (50 vp/cell), did not exhibit detectable cell killing activity. Lovastatin (2.5 μM) alone induced a minor level of *in vitro* killing. AdE4 (50 vp/cell) alone exhibited some cell killing activity, compared to DMSO control. Lovastatin (2.5 μM) significantly increased cell killing activity of AdE4 (50 vp/cell) (P<0.01). AdE4-TRAIL exhibited more cell killing activity than AdE4 (P<0.01), and lovastatin enhanced this even more when used with AdE4-TRAIL (P<0.01). Together, these *in vitro* results show that lovastatin significantly enhanced the cell killing activity of AdE4, and further enhanced cell killing induced by AdE4-TRAIL.

We tested the extent of apoptosis induced by lovastatin and adenovirus constructs in CWR22rv and C4-2 cells. Cells were treated with lovastatin for 16 hours, followed by virus infection for 24 hours. As shown in Figure 2B, in CWR22rv cells, AdE4(ΔTATA)(100 vp/cell) induced minor cell apoptosis (12.2%). Lovastatin (5 μM) did not significantly affect AdE4(ΔTATA)-induced apoptosis (16.3%). AdE4 (100 vp/cell) induced more cell apoptosis than AdE4(ΔTATA) (25.1%). Lovastatin (5 μM) significantly increased AdE4 (100 vp/cell)-induced cell apoptosis (43.6%) (P<0.01). AdE4-TRAIL exhibited a stronger apoptosis induction effect than AdE4 (40.2%, P<0.01); lovastatin further enhanced cell apoptosis of AdE4-TRAIL (62.4%, P<0.01). In C4-2 cells, AdE4(ΔTATA)(50 vp/cell) induced minor cell apoptosis (14.5%), while lovastatin (2.5 μM) did not significantly increase AdE4(ΔTATA)-induced apoptosis (15.3%). AdE4 (50 vp/cell) induced more cell apoptosis than AdE4(ΔTATA) (28.2%, P<0.01). Lovastatin (2.5 μM) significantly increased AdE4 (50 vp/cell)-induced cell apoptosis (37.4%, P<0.01). AdE4-TRAIL exhibited a stronger apoptosis induction response than AdE4 (45.2%, P<0.01). Lovastatin further enhanced the cell apoptosis induced by AdE4-TRAIL (64.7%, P<0.01, Figure 2B).

### Lovastatin significantly elevated viral transduction efficiency and AdE4-delivered transgene expression

**Figure 3:**
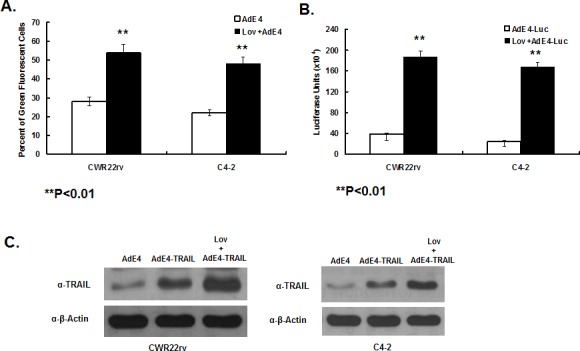
Lovastatin significantly enhanced AdE4-delivered transgene expression CWR22rv and C4-2 cells were treated with vehicle (DMSO) or lovastatin at 10 μM for 16 hours. Then cells were infected with 100 vp/cell of AdE4 (A.) or AdE4-Luc (B.) or AdE4-TRAIL (C.) for the next 48 hours. AdE4-infected green fluorescent cells were monitored by FACS (A.); luciferase activity was measured by a luminometer (B.); TRAIL protein expression was assessed by western blotting (C.), all as described in Methods.

In order to investigate the mechanism by which lovastatin enhances the *in vivo* antitumor effect of AdE4, *in vitro* cell killing and apoptosis induction, we first tested the effect of lovastatin on viral transduction efficiency and AdE4-delivered transgene expression. CWR22rv and C4-2 cells were pre-treated with lovastatin, followed by AdE4 infection for 24 hours. AdE4 vector encodes the EGFP gene, thereby allowing viral transduction efficiency to be assessed using flow cytometry to measure the percentage of green fluorescent cells. Compared to AdE4 alone, lovastatin increased the percent of green fluorescent cells by 23% or 27%, respectively, in CWR22rv or C4-2 cells (P<0.01, Figure 3A). To confirm this result, we infected the cells with AdE4-Luc, a PRRA vector expressing luciferase gene (Figure 1A). Luciferase activity was used as a measure of viral transduction efficiency, and measuring with a luminometer. Consistent with its effects in the AdE4 experiment, lovastatin significantly increased luciferase activity by 4~5-fold in both cell lines (P<0.01, Figure 3B).

We also tested TRAIL protein expression after CWR22rv and C4-2 cells were pre-treated with lovastatin, and then infected with AdE4 or AdE4-TRAIL for 48 hours. TRAIL protein expression was detected by western blot. Lovastatin significantly increased AdE4-mediated TRAIL protein expression (Figure 3C).

### Lovastatin enhanced viral binding, internalization and trafficking to nuclei

**Figure 4:**
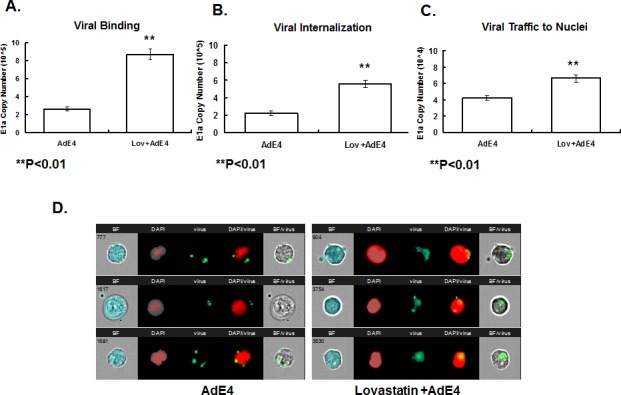
Lovastatin significantly enhanced adenoviral binding, internalization and intercellular trafficking to the nuclei A. CWR22rv cells were treated with vehicle (DMSO) or lovastatin at 10 μM for 16 hours, then exposed to 5000 vp/cell of AdE4 at 4ºC for 60 minutes. The unbound viral particles were removed; DNA of bound virus particles was processed for analysis of adenovirus *E1a* copy number by quantitative PCR assay. B. After viral binding, cells were allowed to internalize virus particles at 37ºC for other 30 minutes. The attached but uninternalized viral particles were removed, and the DNA of internalized adenoviral particles for adenoviral *E1a* copy number using quantitative PCR. C. Nuclear DNA was separated, and adenovirus *E1a* copy number inside the nuclei was analyzed by quantitative PCR. D. Screen views of virus trafficking in individual cells, as monitored by the Amnis Imagestream^X^ cell analyzer. Adenoviral particles were labeled with Alexa Fluor® 488 dye. CWR22rv cells were treated with vehicle (DMSO) or lovastatin at 10 μM for 16 hours, and then exposed to virus at 5000 vp/cell of dye-labeled AdE4 for 60 minutes at 37ºC, to allow virus internalization; and then for 30 minutes, to allow for virus trafficking to the nuclei. Nuclei were stained with DAPI. Cells and viral particles were monitored using INSPIRE™ software, and the co-localization data of viral particles and nuclei were analyzed using IDEAS software. Three representative AdE4-treated single cells are shown in the left panel, and 3 representative AdE4 plus lovastatin-treated single cells are shown in the right panel. BF: brightfield showing the cell morphology; DAPI: nuclei; virus: dye-stained viral particles; DAPI/virus: co-localization of virus and nuclei showing viruses inside the nuclei; BF/virus: co-localization of virus and cell showing viruses inside cells.

The adenoviral infection pathway includes several critical steps: [1] Binding of adenovirus 5 capsid to cell receptor CAR. [2] Internalization by endocytosis through integrin α_v,_ β_1_ and β_3_. [3] Lysis of the endosomal membrane, resulting in escape to the cytosol to facilitate trafficking along microtubules. [4] Binding to the nuclear envelop to enable insertion of the viral genome through nuclear pores. To test the impact of lovastatin on AdE4 binding, internalization and trafficking to nuclei, viral particles were continuously tracked after treating CWR22rv cells with lovastatin, and then infecting with AdE4 infection. The cells were incubated with adenoviral particles at 4ºC for 60 mins. After PBS washing, the amount of adenoviral particles was determined by assessing adenoviral *E1a* copy number, using quantitative PCR. Then, cells were incubated at 37ºC for another 30 minutes to allow time for virus internalization and trafficking to nuclei to occur. The attached but uninternalized viral particles were removed by subtisilin. Internalized adenoviral particles were analyzed for adenoviral *E1a* copy number by quantitative PCR assays. Nuclear DNA was prepared so that adenoviral *E1a* copy number inside nuclei could be analyzed by quantitative PCR. As shown in Figure 4A, 4B and 4C, lovastatin significantly enhanced AdE4 viral binding, internalization and trafficking to nuclei. Viral copy number increased by approximately 3.5-fold in the viral binding assay, by approximately 2.5-fold in the viral internalization assay, and by 1.6-fold in the assay to assess viral trafficking to nuclei. These results suggest that lovastatin significantly enhanced adenoviral binding, while exhibiting lesser effects on viral internalization and trafficking to nuclei. We speculate that the increased viral copy number in viral internalization and trafficking to nuclei can probably be attributed to the increased viral binding.

To confirm that lovastatin enhanced viral trafficking to nuclei, we monitored viral particles inside the cells. The capsids of AdE4 first were chemically labeled with Alexa Fluor® 488 dye, prior to virus infection. The intracellular virions inside the cytoplasm and nuclei were assessed using an Amnis ImageStream^X^ cell analyzer. As shown in Figure 4D, at 30 minutes after allowing virus to bind at 37ºC, AdE4 viral particles were detected in cytoplasm, but few were detected inside the nuclei. Lovastatin significantly enhanced the adenovirus intracellular trafficking to nuclei, as most of the fluorescence in treated cells was detected inside the nuclei (Figure 4D).

### Lovastatin increased the replication activity of AdE4

To test if lovastatin increases the replication activity of AdE4, we performed an adenovirus replication assay. As described in Table 1, we pre-treated C4-2 or CWR22rv cells with lovastatin at 10 μM, and then infected cells with AdE4 at the dose of 6.6 × 10^4^ virus particles in C4-2 cells and 2 × 10^4^ virus particles in CWR22rv (the virus doses used to infect cells were based on the relative virus infection efficiency of these two cell lines). The media were changed 6 hours after administering virus; 2 days later, supernatants were harvested to assess virus titer. The amount of adenovirus produced was expressed as TCID50. Lovastatin increased the virus titer from 2.5 × 10^6^ to 1.5 × 10^7^ TCID50 in C4-2 cells and from 8 × 10^6^ to 2.5 × 10^7^ TCID50 in CWR22rv cells. These data show that pre-treatment of lovastatin significantly promoted the replication activity of AdE4 in PSA/PSMA-positive cells.

**Table 1:** Impact of Lovastatin on the Replication of AdE4

	Treatment	Input Doses *^a^* (v.p.)	Output Doses *^b^* (TCID50*)
C4-2	AdE4	6.6 × 10^4^	2.5 × 10^6^
	AdE4+Lov (10μM)	6.6 × 10^4^	1.5 × 10^7^
CWR22rv	AdE4	2.0 × 10^4^	8.0 ×10^6^
	AdE4+Lov (10μM)	2.0 × 10^4^	2.5 × 10^7^

Cells were infected with AdE4 for 48 hours, and the cells were frozen/thawed for three times. The soup is used for titer assay.

a Input viral doses mean the virus doses used to infect cells.

b Output viral doses mean the titered virus doses in titer assay.

* The virus production was shown as a TCID50 value (the dilution factor that caused a cytopathic effect in at least four of eight wells of cells in a row on a 96-well plate on day 7)

### Correlation of lovastatin-induced cell apoptosis with cholesterol level in lipid rafts on cell membranes

**Figure 5:**
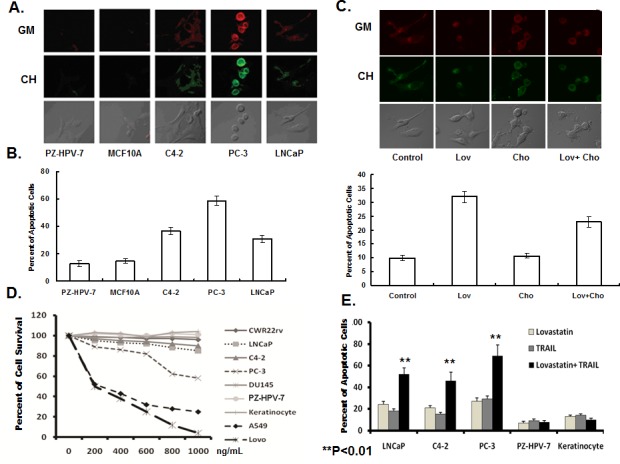
Lovastatin induced cell apoptosis, and sensitized cancer cells to TRAIL-induced apoptosis A. Representative cells to enable visualization and overlays of lipid rafts/cholesterol in non-malignant and cancer cell lines, using confocal microscopy. Non-malignant cells PZ-HPV-7 and MCF10A and prostate cancer cells C4-2, PC-3 and LNCaP were labeled with Alexa red Fluo555/565 – CTXB (lipid rafts, glycolipoprotein microdomains, GM) and green filipin (cholesterol, CH) and analyzed by confocal microscopy. B. The correlation of level of lipid rafts/cholesterol and the sensitivity of cells to lovastatin-induced apoptosis. Serum-starved cells were treated with lovastatin at 10 μM or DSMO control for 16 hours, with triplicate-wells for each cell line. Apoptotic cells were stained with Annexin V-FITC and PI, and detected by flow cytometry. Data were expressed as the ratio of lovastatin-treated cells to control. C. The effect of lovastatin on the level of cholesterol in the lipid rafts and apoptosis in prostate cancer cells. Serum-starved cells were treated with DSMO (control) or lovastatin at 10 μM for 16 hours, and then incubated with or without 500 μM cholesterol for 2 hours. Cells were stained with CTXB-Alexa 555/558 (GM) and filipin (CH), and monitored by confocal microscopy. The cells were processed for Annexin V-FITC and PI staining, and the percent apoptotic cells analyzed by flow cytometry. Each experiment was replicated 3 times. D. Prostate cancer cells are resistant to TRAIL-induced apoptosis. Prostate cancer cells LNCap, C4-2, CWR22rv, PC-3, DU145, non-malignant prostate epithelial cells PZ-HPV-7, keratinocytes, non small lung adenocarcinoma cells A549 and colon cancer Lovo cells were treated with TRAIL protein for 24 hours at a range of doses (n=5 for each cell line). Cell viability was measured by MTT assay at 72 hours after drug treatment. E. Lovastatin significantly enhanced TRAIL-induced apoptosis in prostate cancer cells, but not in normal cells. LNCaP, C4-2, PC-3, PZ-HPV-7 and keratinocytes were treated with lovastatin at 10 μM for 16 hours, before being treated with or without TRAIL(200 ng/mL) for 24 hours (n=4/group). The cells were processed for Annexin V-FITC and PI staining, and the percent apoptotic cells analyzed by flow cytometry.

Non-malignant cells PZ-HPV-7 and MCF10A, and PCa cells C4-2, PC-3 and LNCaP cells were used to test the efficacy of lovastatin on cell apoptosis. Lovastatin (10 μM) selectively induced cell apoptosis in PCa cells, but not in non-malignant cells. We labeled lipid rafts on the cellular membrane with Alexa Fluo555/565-CTXB (GM), and labeled cholesterol with filipin (CH). The levels of lipid rafts and cholesterol on the membrane of PCa cells were much higher in PCa cells, than in non-malignant cells. Lipid raft levels were highest in PC-3 cells. Interestingly, the level of lipid rafts and cholesterol inversely correlated with the sensitivity of cells to lovastatin-induced apoptosis. We found that the higher the level of lipid rafts and cholesterol, the greater the magnitude of lovastatin-induced apoptosis (Figure 5A).

Because Lovastatin is a cholesterol-lowering drug, we tested the hypothesis that induction of apoptosis by lovastatin is via depletion of cholesterol in lipid rafts. We treated C4-2 cells with 10 μM lovastatin alone for 16 hours, or with 500 μM cholesterol alone for 2 hours, or 10 μM lovastatin for 16 hours, and followed by 500 μM cholesterol for 2 hours. Lovastatin significantly decreased cholesterol and lipid raft levels on cell membranes. In contrast, addition of cholesterol elevated the cholesterol level in lipid rafts. The lovastatin depletion of cholesterol and lipid rafts could be reversed by the addition of cholesterol (Figure 5B). The levels of cholesterol and lipid rafts on cell membranes closely correlated with apoptosis induction by lovastatin. In C4-2 cells, lovastatin (10 μM) increased cell apoptosis from 9.8% to 32.4%, while cholesterol did not significantly alter cell apoptosis. The addition of cholesterol reduced lovastatin-induced cell apoptosis from 32.4% to 23.6%. Based on the correlation between induction of cell apoptosis by lovastatin and lowered levels of cholesterol and lipid rafts on the cell membranes, we speculate that the effect on apoptosis may be affected via depletion of cholesterol in the lipid rafts.

### Lovastatin sensitizes PCa cells to TRAIL-induced apoptosis

TNF-related apoptosis-inducing ligand (TRAIL) induces cell apoptosis by binding to the death receptors, DR4 and DR5 [11]. As shown in Figure 5C, A549, a lung adenocarcinoma cell line and Lovo, a colorectal cancer cell line exhibited high sensitivity to TRAIL-induced apoptosis, while PC-3, a metastatic PCa cell line, showed some sensitivity to TRAIL-induced apoptosis. Other PCa cell lines, CWR22rv, LNCaP and C4-2, and non-malignant PZ-HPV-7 cells and keratinocytes were resistant to TRAIL-induced apoptosis (Figure 5C). In the PCa cell lines, LNCaP, C4-2 and PC-3, lovastatin (10 μM) or TRAIL(200 ng/mL) alone induced cell apoptosis. The percent apoptotic cells further increased when LNCaP, C4-2 and PC-3 cells were treated with a combination of lovastatin and TRAIL, but did not change in control PZ-HPV-7 cells or keratinocytes (Figure 5D). These data indicated that lovastatin selectively sensitized TRAIL-induced apoptosis in cancer cells, but had no further effect in non-malignant cell lines.

### Lovastatin increased expression of adenovirus binding receptors and TRAIL receptors in PCa cells

**Figure 6:**
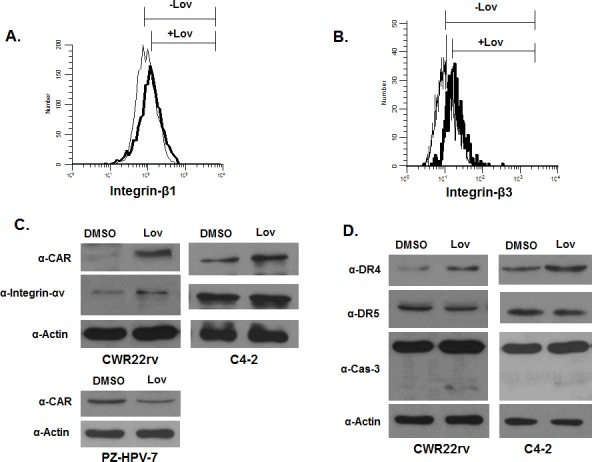

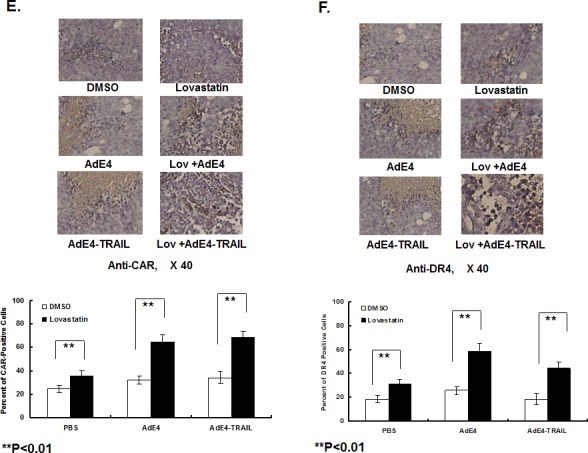
A to D. Lovastatin increased protein expression of selected cell receptors, which included CAR and integrin αv, β1 and β3, and TRAIL receptors DR4. CWR22rv, C4-2 or PZ-HPV-7 cells were treated with DSMO (control) or lovastatin at 10 μM for 16 hours. Protein expression of integrin β1 and β3 were determined in CWR22rv cells by flow cytometry (A, B); expression of CAR, integrin αν, DR4, DR5 and cleaved caspase 3 were determined in CWR22rv and C4-2 cells by western blotting (C, D). Protein expression of CAR in PZ-HPV-7 cells is shown in C. E. and F. Lovastatin significantly enhanced CAR and DR4 expression inside xenograft tumor tissues of mice after 28 days of treatment. CAR and DR4 expression in tissue sections of xenografts were evaluated by immunohistochemical staining. The number of positive cells was counted in 10 randomly selected vision fields (×40) for each tissue section; 3 tissue sections were averaged to represent each tumor.

Adenovirus 5 infects cells by binding to CAR receptor, and internalizing into cells via the integrins [24]. The CAR receptors and integrins that bind adenovirus reside in cholesterol-enriched lipid rafts. Their expression is closely associated with the status of lipid rafts, and is affected by modification of lipid rafts [25-27]. Because lovastatin depletes cholesterol level and affects lipid rafts, we speculated that lovastatin might increase expression of CAR, integrin α_v,_ β_1_and β_3,_ to enhance AdE4 binding and internalization. We tested the effect of lovastatin on expression levels of CAR and integrins α_v,_ β_1_ and β_3_ on the cell surface of CWR22rv and C4-2 cells, by either flow cytometry or western blot. As we hypothesized, lovastatin significantly increased the expression of CAR, consistent with increased adenovirus binding (Figure 6C). Lovastatin also slightly increased the expression of integrin β_1_ and β_3_ (Figure 6A and 6B) and integrin α_v_ (Figure 6C), consistent with enhanced virus internalization. These data are consistent with our findings of changes in viral copy number after virus infection. Lovastatin enhanced adenoviral binding, internalization and viral trafficking to nuclei (Figure 4A, 4B and 4C). These data suggest that lovastatin may enhance AdE4 infection efficiency and virus-delivered transgene levels, in part, by increasing the expression of adenovirus binding receptor CAR. Additional viral binding promotes viral internalization and trafficking to nuclei to increase virus replication and apoptotic cell death.

We also assessed the effect of lovastatin on CAR expression in control non-malignant PZ-HPV-7 cells. Interestingly, lovastatin affected CAR expression differently in normal PZ-HPV-7, compared to the cancer cell lines. In CWR22rv and C4-2, which exhibit high cholesterol levels in lipid rafts, lovastatin increased CAR expression by depleting cholesterol in lipid rafts. In the non-malignant cell lines, such as PZ-HPV-7 that exhibit low cholesterol levels, lovastatin decreased CAR expression (Figure 5A and Figure 6C).

Previous studies demonstrated that death receptors, such as DR4 and DR5, are recruited into lipid rafts to facilitate protein-protein interactions and apoptosis induction [28]. Translocation of DR4/DR5 to lipid rafts promoted TRAIL-induced apoptosis [29]. When there is lower DR4/DR5 expression in lipid rafts, cells exhibit more resistance to TRAIL-induced apoptosis [28]. We hypothesized that, because lovastatin depleted cholesterol in the lipid rafts, lovastatin may sensitize cells to TRAIL-induced apoptosis by regulating DR4 and DR5 expression. We tested the effect of lovastatin on DR4 and DR5 expression at the cell surface of CWR22rv and C4-2 cells, using western blot. Lovastatin significantly increased DR4 expression, but not DR5 expression, and induced caspase-3-mediated apoptosis (Figure 6D).

We tested the protein expression of CAR and DR4 by using IHC staining of tumor tissues harvested during the *in vivo* studies. Lovastatin alone increased CAR expression compared to the DMSO control. Lovastatin significantly increased CAR expression inside tumor tissues when combined with AdE4 or AdE4-TRAIL, compared to either AdE4 or AdE4-TRAIL alone (Figure 6E). These results from *in vivo* experiments confirm and extend our *in vitro* results. Lovastatin increased DR4 expression inside tumor tissues, compared to the DMSO control. Adenovirus infection slightly increased DR4 expression, while lovastatin combined with AdE4 significantly increased DR4 expression inside tumor tissues (P<0.01). In contrast, AdE4-TRAIL decreased DR4 expression, probably because of the neutralization of DR4 by TRAIL ligand. DR4 expression also decreased in tumors treated with lovastatin and AdE4-TRAIL compared to those treated with lovastatin and AdE4 (Figure 6F).

## DISCUSSION

In the present report, we explored the efficacy of combining lovastatin with PRRA-mediated TRAIL in preclinical experiments, as a novel strategy to treat refractory PCa. The combination enhanced antitumor efficacy *in vivo* and *in vitro* through activation of multiple molecular mechanisms. Lovostatin enhanced the infection efficiency of PRRA and virus-delivered transgene expression by significantly increasing the expression level of CAR, and slightly increasing the expression of integrins. Lovastatin enhanced TRAIL-induced apoptosis by increasing the expression of death receptor DR4. Interestingly, these effects of lovastatin on CAR, integrins and DR4 expression levels were closely associated with lovastatin-depletion of cholesterol in lipid rafts of cellular membrane.

Lipid raft microdomains reside in plasma membrane of cells, and they consist of dynamic assemblies of cholesterol [30]. Lipids localize to the exoplasmic leaflet of the membrane bilayer, while cholesterol is found in the inner leaflet [31]. Upon activation of key receptors by ligand binding, lipid rafts cluster into larger macrodomains. Various signaling molecules are recruited into these lipid raft macrodomains, to form the signaling platforms for transmembrane signal transduction [30-32]. Signaling molecules include those associated with virus infection signaling [33], apoptotic pathway signaling [34], death receptor activation signaling [32], and carcinogenesis [35]. Signal transduction can be affected by modifying the cholesterol content of lipid rafts. The level of cholesterol in lipid rafts was significantly higher in cancer cells than normal cells, and cholesterol accumulation enhanced cancer transformation and progression [36, 37]. Statins, such as lovastatin [38, 39] and simvastatin [37, 40], which inhibit the enzyme HMG-CoA reductase, catalyze a key rate-limiting step in cholesterol biosynthesis, and induce cell apoptosis by inhibiting PI3K/Akt signaling.

The adenovirus 5 binding receptor, CAR, and integrins are found in cholesterol-enriched lipid rafts [25-27]. Their expression is closely associated with the status of lipid rafts, such that modification of lipid rafts affects their expression [25-27]. CAR receptors and integrins, are critical for adenovirus 5 binding and internalization in cells. In the current study, lovastatin significantly increased the expression level of CAR, and slightly increased the expression levels of integrins through cholesterol-depletion in the lipid rafts of PCa cells. In contrast, lovastatin decreased CAR expression in non-malignant cell lines with low cholesterol content. The results are consistent with the recent report that lovastatin decreased CAR expression in human normal umbilical vein endothelial cells (HUVEC) [41]. We speculate that the effect of lovastatin on CAR expression may be selectively governed by the availability and level of cholesterol in lipid rafts of cancer cells.

Lipid rafts are closely associated with the activation of death receptors, such as DR4 [42], DR5 [42, 43] and FAS [44], depending on the cell model system. TRAIL-induced apoptosis is associated with lipid raft formation and the status of death receptors inside the raft [45]. We report for the first time that induction of apoptosis by lovastatin correlated with the cholesterol level of lipid rafts, and that apoptosis induced by lovastatin *in vitro* can be mitigated by the addition of cholesterol. We speculate that, by increasing death receptor expression, lovastatin may overcome cancer cell resistance to TRAIL.

These studies, for the first time, demonstrate the correlations between cholesterol/lipid rafts and oncolytic adenovirus infection efficiency and antitumor efficacy of TRAIL *in vitro*. These findings add to our understanding of the molecular mechanisms that support the use of lovastatin, in combination with PRRA-TRAIL, as a novel experimental treatment strategy for refractory PCa in the future.

## MATERIALS AND METHODS

### *In vivo* animal studies

A subcutaneous tumor xenograft model using the CWR22rv cell line was established as described previously [4]. Mice were inoculated with CWR22rv cells (1× 10^6^ in 100 mL culture media) by subcutaneous injection. When tumor size reached ~5 mm in diameter at 2-3 weeks after cell inoculation, the mice were randomly assigned to 6 groups. Three groups, received 10 mg/kg/day of lovastatin in DMSO, and another 3 groups, served as controls, received PBS with the same dose of DMSO daily ip injections for 7 days. After the 7 day period, mice in each three groups individually received intratumor (i.t.) injections of 2×10^7^ infectious unit (IFU) of AdE4PSESE1a (AdE4), AdE4-TRAIL or PBS control. To clarify, mice were sorted into the following groups (n=8 each group): [1] DMSO (i.p.), [2] lovastatin (i.p.), [3] AdE4PSESE1a (AdE4, i.t.), [4] AdE4-TRAIL (i.t.), [5] lovastatin (i.p.) with AdE4 (i.t.), and [6] lovastatin (i.p.) with AdE4-TRAIL (i.t.). Tumor size was measured with calipers on days 3, 7, 14, 21 and 28, and tumor volume was calculated as length × width^2^ × 0.5236 [45]. Mice were sacrificed when tumor size exceeded 1000 mm^3^, or on day 28 if tumor size did not reach 1000 mm^3^.

### Histology, immunohistochemistry and in situ Terminal deoxynucleotidyl transferase dUTP nick end labeling (TUNEL) assay

After mice were sacrificed, tumors were collected and immediately fixed in 10% phosphate buffered formalin, processed, embedded in paraffin and cut into 5 um thin histological sections. To assess adenovirus infection, mouse monoclonal (SPM 230) antibody (ready for use) against adenovirus type 5 E1a (Abcam, Cambridge, MA, USA) and a super sensitive biotinylated second mouse antibody (BioGenex, San Francisco, CA, USA) were used. To assess expression of CAR and DR4 protein, antibodies against CAR (H-300) or DR4 (C-20) (Santa Cruz Biotechnology. Santa Cruz, CA, USA) were used. Assessment of *in situ* apoptosis was performed with the TUNEL assay, using the manufacturer's instructions (Roche Dianostics, Indianapolis, IN, USA). Positive-staining cells were counted in 10 randomly selected vision fields (× 200) in each tissue slide. We counted cells in 3 slides for each tumor. Results were expressed as mean ± standard deviation of 30 measurements/tumor.

### Cell lines and cell culture

PSA/PSMA positive PCa cell lines, LNCaP, C4-2, CWR22rv and negative PCa cell lines PC3 and DU145 (ATCC, Manassas, VA, USA), were each maintained in RPMI-1640 medium, supplemented with 10% FBS and 1% penicillin/streptomycin. Non small lung adenocarcinoma cell line, A549, and the Lovo colon cancer cell line (ATCC), were maintained in DMEM medium, supplemented with 10% FBS and 1% penicillin/streptomycin. Immortalized, non-malignant breast epithelial cell line, MCF10A, was maintained in the growth media as previously reported [46]. Non-malignant, prostate epithelial cell line, PZ-HPV-7 (ATCC), and keratinocytes (Lonza, Anaheim, CA, USA) were maintained in KGM-Gold Bullet Kit (containing KBM-Gold Basal Medium plus KGM-Gold SingleQuot Kit).

### Cell Killing Assay

C4-2 and CWR22rv cells were each seeded onto 24-well plates (1 × 10^5^/well), and treated with lovastatin (10 uM), AdE4(ΔTATA)(replication-deficient adenovirus), AdE4 or AdE4-TRAIL at 100 virus particle (vp)/cell, or lovastatin (10 uM) for 16 hours, and followed by virus infection of AdE4(ΔTATA), AdE4 or AdE4-TRAIL. Four wells were used for each treatment. All cells were stained with crystal violet dye at 5 days after virus infection, and lysed for OD read at 590 nm.

### Cell Viability Assay

LNCap, C4-2, CWR22rv, PC-3, DU145, PZ-HPV-7, keratinocytes, A549 and Lovo cells were each treated with TRAIL protein (R&D, Minneapolis, MN, USA) at a range of doses. Cell viability was measured by MTT assay 72 hours after drug treatment.

### Flow Cytometry Analysis to detect Cell Apoptosis

CWR22rv and C4-2 cells were each seeded onto 12-well plates (2.5 × 10^5^/well), and treated with either vehicle control (DMSO), or 10 μM lovastatin, for 16 hours before infection with AdE4(ΔTATA)(replication-deficient), AdE4 or AdE4-TRAIL, at 100 vp/cell (n=3 wells/group). Cells were harvested with 0.25% trypsin 48 hours post-virus infection, and washed once with PBS preparatory to apoptosis detection. Serum-starved PZ-HPV-7 and MCF10A and C4-2, PC-3 and LNCaP cells were treated with 10 μM lovastatin for 24 hours, using triplicate-wells for each cell line. Serum-starved LNCaP cells were treated with or without 10 μM lovastatin for 16 hours, and then incubated with or without 500 μM cholesterol for 2 hours. LNCaP, C4-2, PC-3, and PZ-HPV-7 and keratinocytes were treated with or without 10 μM lovastatin for 16 hours, followed by the treatment of 200 ng/mL of TRAIL protein (R&D) for another 24 hours. Apoptotic cells were stained with Annexin V-FITC and PI, and detected by flow cytometry.

### Viral Replication Assay

C4-2 and CWR22rv cells were seeded onto 6-well plates (1 × 10^6^/well), and treated with 10 μM lovastatin for 16 hours. Then cells were infected with AdE4 at 6.6 × 10^4^ vp in C4-2 and 2 × 10^4^ vp in CWR22rv cells to ensure equivalent viral infection efficiency. The doses for these cell lines were identified in preliminary experiments. The media were changed 6 hours after virus infection. The cells were harvested, and subjected to 3 freeze/thaw cycles 48 hours post-virus infection. Virus soup was harvested, and titrated using virus titer assay described in previous publications, with the amount of produced adenovirus was expressed as TCID50 [4, 8].

### Fluorescence Labeling of Cells for Lipid Raft and Cholesterol Detection

Cells were grown on chamber slides (Nalgen Nunc International, Monroe County, NY, USA), and fixed in 3% paraformaldehyde for 1 h at room temperature, before being incubated with 1 ml of 1.5 mg glycine/ml PBS for 10 min at room temperature, to quench fluorescence due to paraformaldehyde. The cells were then labeled with Alexa Fluo555/565-CTXB (0.5 μg/ml, Molecular Probes of Life Technology, Grand Island, NY, USA) for 10 minutes on ice. After rinsing in PBS, the cells were incubated with 1 ml of Filipin working solution (0.05 mg/ml in PBS, Sigma Aldrich, St. Louis, MO, USA) for 2 h at room temperature. Using confocal microscopy, glycolipoprotein microdomains (GM, lipid rafts) were viewed first, using a 543 nm laser, and then cholesterol was viewed, using a UV 350nm laser.

### Virus Binding and Intercellular Trafficking Detection by PCR Assays

The experiment protocol was developed according to Wang, et al's report with modification [47]. Suspended CWR22rv cells were incubated with AdE4 (5000vp/cell) at 4 ºC, with vigorous shaking for 60 minutes, and the unbound viral particles were removed by washing cells in cold PBS 3 times. The DNA of bound virus particles was processed for analysis of adenovirus *E1a* copy number by quantitative PCR assay. After viral binding, virus particles were allowed to internalize and traffic to nuclei, at 37ºC for 30 minutes. To assess viral internalization, the attached but uninternalized viral particles were first removed using subtisilin (2 mg/mL, Sigma Aldrich), before assessing internalized adenoviral particles for adenoviral *E1a* copy number using quantitative PCR. Next, nuclear DNA was isolated using the NE-PER nuclear and cytoplasmic kit (Pierce Biotechnology, Ockford, IL, USA) adenovirus *E1a* copy number in the nuclei was analyzed by quantitative PCR assay.

### Virus Binding and Intercellular Trafficking Detection by Imagestream Cell Analyzer

AdE4 virus particles were labeled with Alexa Fluor® 488 dye. Next, suspended CWR22rv cells were incubated with AdE4 (5000 vp/cell) at 4 ºC, with vigorous shaking for 60 minutes [47]. Cells were then cultured at 37 ºC for 30 minutes. After brief staining with DAPI, the cells were entered into the Amnis ImageStream^X^ cell analyzer (Amnis Corporation, Seattle, WA, USA) using low flow rate/high sensitivity settings, and evaluated using INSPIRE™ software. Amnis ImageStream Analyzer and IDEAS Analysis Software represent advances in multispectral imaging technology, because cell morphology and fluorescent labeling of genes or proteins can be simultaneously visualized in single cells. This powerful combination of quantitative image analysis and flow cytometry in a single platform creates exceptional new experimental capabilities [48]. The instrument and INSPIRE™ software were set up as follows: Channel 1(DAPI); Channel 2 (virus, Alexa Fluor® 488); Channel 3 (brightfield); and Channel 6 (scattering channel). Magnification was set at 60×, providing a pixel size of 0.33 microns. The 405 nm and 488 nm lasers were used to activate multispectral fluorescence. The flow rate was set to low speed/high sensitivity; stream alignment was adjusted as necessary. The co-localization of virus and cellular nuclei, or virus and cells in brightfield was analyzed by IDEAS software.

### Preparation of Cells for Flow Cytometry Analyses to detect GFP Transgene Expression

CWR22rv and C4-2 cells were seeded onto 12-well plates (2.5 × 10^5^ cells per well), and treated with 10 μM lovastatin for 16 hours before infection with AdE4 at 100 vp/cell. DMSO-treated cells were used as the control. Cells were harvested using 0.25% trypsin 24 hours post-infection, washed with FACS buffer (PBS with 5% FBS and 0.1% sodium azide) on ice, and then fixed in 0.5 ml of cold 1% paraformaldehyde solution. The GFP positive cells were analyzed.by flow cytometry.

### Bioluminescence analysis to detect luciferase transgene expression

CWR22rv and C4-2 cells were seeded onto 12-well plates (2.5 × 10^5^ cells per well), and treated with 10μM lovastatin for 16 hours before being exposed to infection with AdE4-Luc (AdE4 vector encoding luciferase gene) at 100 vp/cell. DMSO-treated cells were used as the control. Cells were lysed 24 hours post-infection, and the cell luciferase activity was assessed with a Glomax luminometer (Promega, Madison, WI, USA).

### Western Blotting

CWR22rv, C4-2 and PZ-HPV-7 cells were seeded onto 12-well plates (2.5 × 10^5^ cells per well), and treated with 10 μM lovastatin for 16 hours before infection with AdE4 at 100 vp/cell. DMSO-treated cells were used as the control. Protein preparations (40 μg) were subjected to SDS-PAGE separation, and electroblotted to a nitrocellulose membrane. Antibodies against human CAR, integrin αυ, DR4, DR5 and caspase 3 were purchased from Santa Cruz Biotechnology. Primary antibodies were detected using horseradish peroxidase-conjugated anti-rabbit IgG secondary antibody (Cell Signaling, Danvers, MA, USA).

### Detection of Integrin β1 and β3 Expression on Cells, Using Flow Cytometry

CWR22rv cells were seeded onto 12-well plates (2.5 × 10^5^/well), and treated with DMSO or lovastatin at 10μM for 16 hours. Cells were harvested using 0.25% trypsin, washed with FACS buffer on ice, re-suspended in 50 μl of buffer, and incubated on ice for 30 minutes with anti-human integrin β1 or β3 (R&D, Minneapolis, MN, USA), followed by washing three times. The cells were then incubated with a fluorescein-conjugated IgG secondary antibody (R&D) for 30 minutes, followed by 3 washes. Finally, cells were fixed in 0.5 ml of cold 1% paraformaldehyde solution in preparation for flow cytometry analysis. The positive cells were analyzed in a histogram data.

### Statistical Analysis

The statistical comparisons of adenovirus 5 E1a, TUNEL, CAR and DR4 positive cells in the tissue sections, and *in vitro* cell killing activity, cell apoptosis, virus-mediated transgene expression, adenoviral binding, internalization and intercellular trafficking, TRAIL cytotoxicity between treatments were carried out by unpaired 2-tailed Student's t test. In xenograft tumor studies, statistical analyses used one-way ANOVA to compare the tumor size among all treatment groups, and the pair-wise comparisons between treatment groups were adjusted with Bonferroni's correlation. All data were presented as mean±SEM, and *P* value of less than 0.05 were considered to be statistically significant for all tests.
